# Supplementary material for: Platycodonis Radix Alleviates LPS-Induced Lung Inflammation through Modulation of TRPA1 Channels
Source: Molecules. 2023 Jul 5;28(13):5213. doi: 10.3390/molecules28135213 (PMC10343319; doi:10.3390/molecules28135213)
Supplement: Supplementary file 1 [file molecules-28-05213-s001.zip › molecules-2468579-supplementary.pdf]

supporting information

1. Five primary platycodins identified in of PR water extract

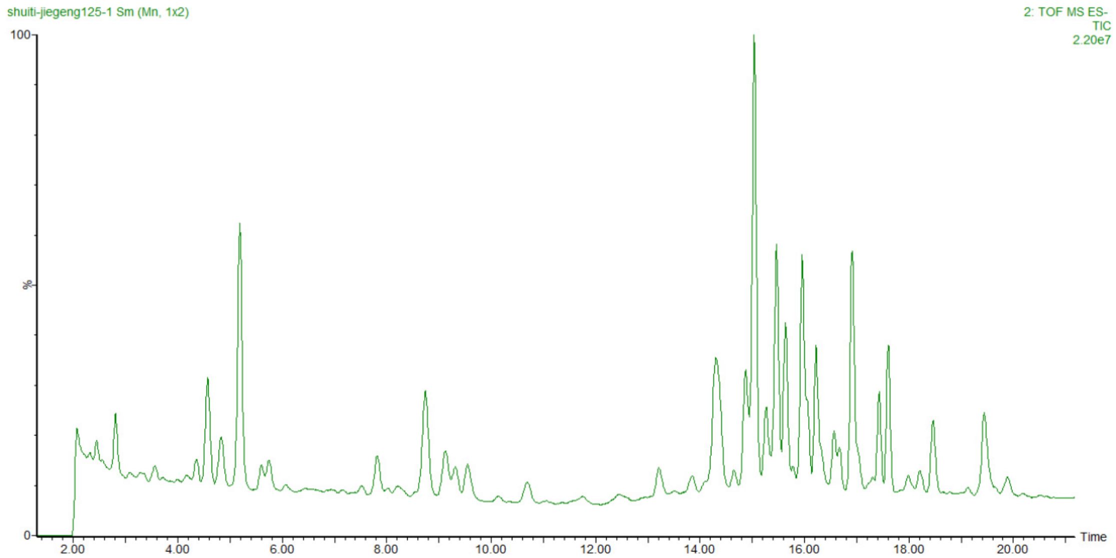

Figure S1 The total ion chromatograms of PR.

A

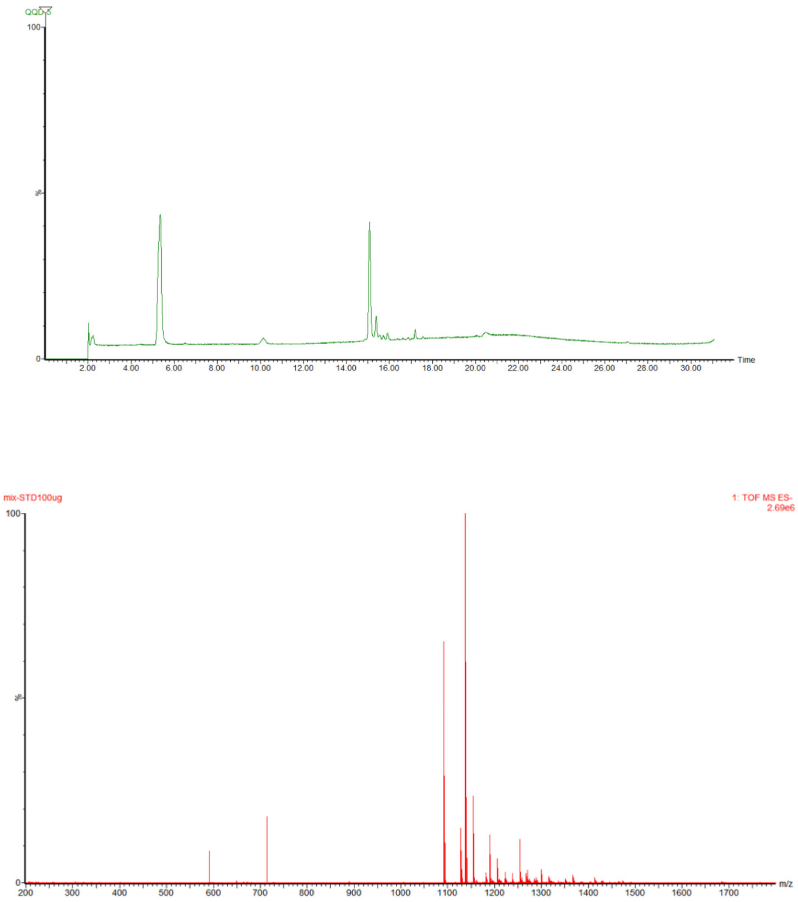

B

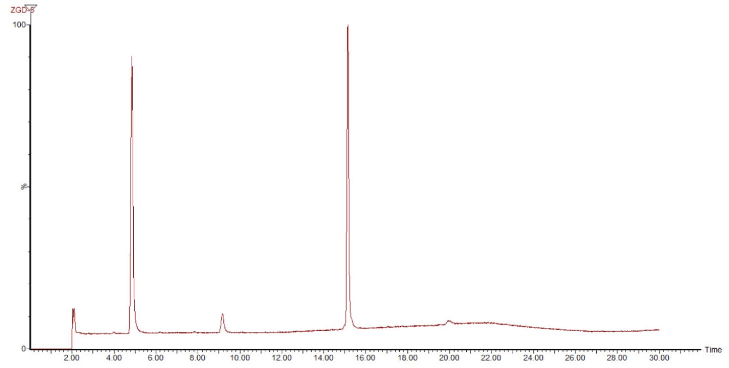
100
%
0
2.00 4.00 6.00 8.00 10.00 12.00 14.00 16.00 18.00 20.00 22.00 24.00 26.00 28.00 30.00
Time

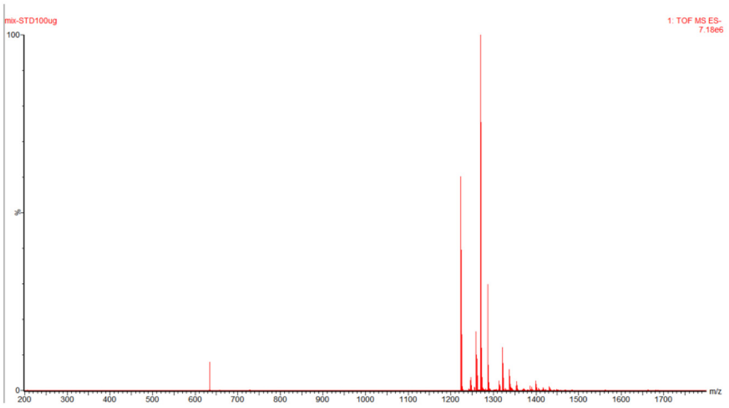
mix-STD100ug
1: TOF MS ES-
7.18e6
100
%
0
200 300 400 500 600 700 800 900 1000 1100 1200 1300 1400 1500 1600 1700
m/z

C

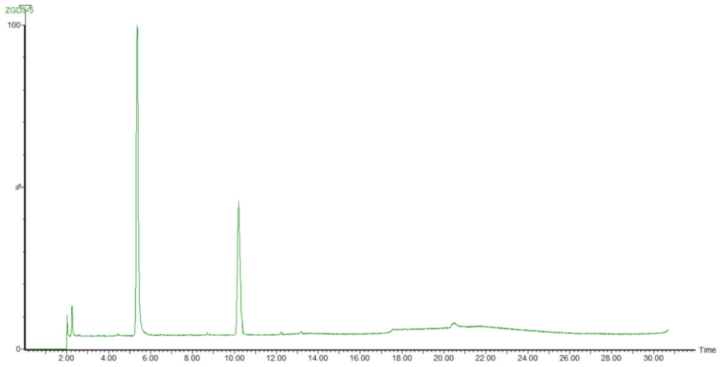
100
%
0
2.00 4.00 6.00 8.00 10.00 12.00 14.00 16.00 18.00 20.00 22.00 24.00 26.00 28.00 30.00
Time

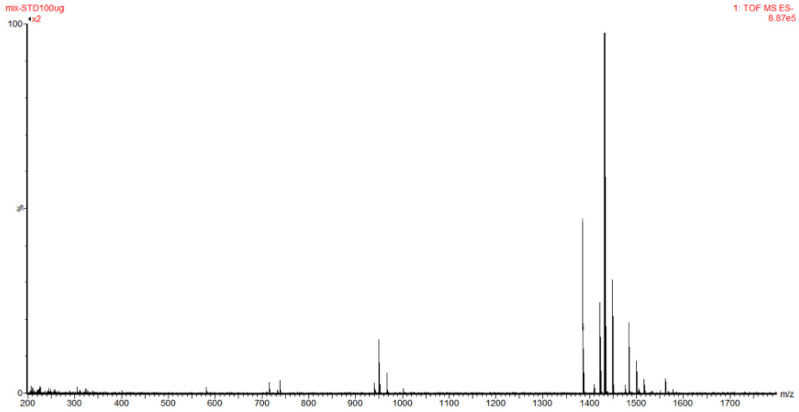
mix-STD100ug
x2
1: TOF MS ES-
8.87e5
100
%
0
200 300 400 500 600 700 800 900 1000 1100 1200 1300 1400 1500 1600 1700
m/z

D

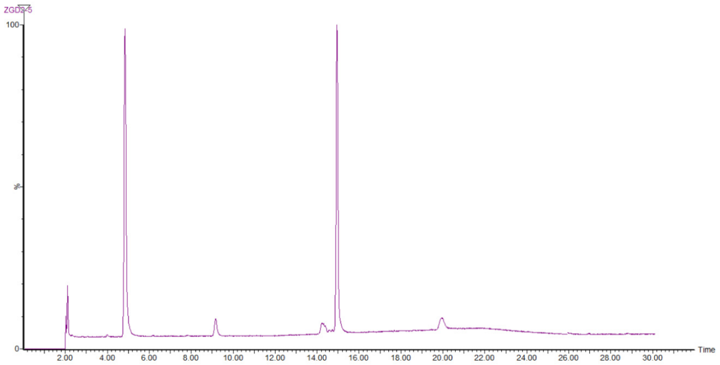
100
%
0
2.00 4.00 6.00 8.00 10.00 12.00 14.00 16.00 18.00 20.00 22.00 24.00 26.00 28.00 30.00
Time

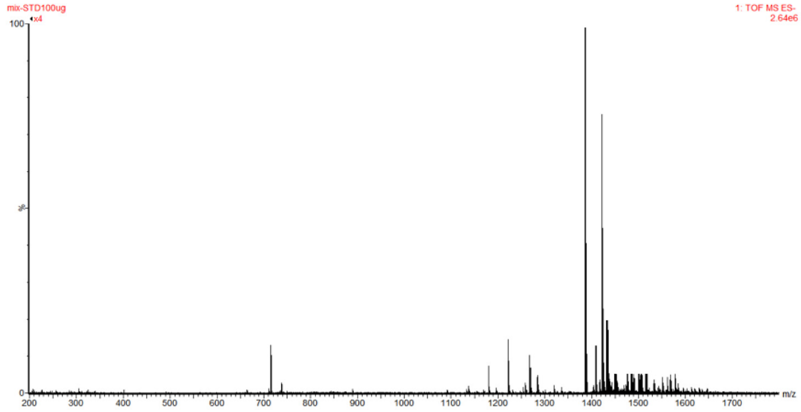
mix-STD100ug
x4
1: TOF MS ES-
2.64e6
100
%
0
200 300 400 500 600 700 800 900 1000 1100 1200 1300 1400 1500 1600 1700
m/z

E

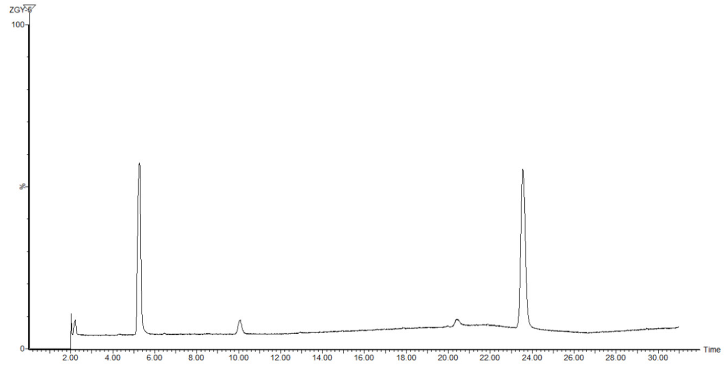

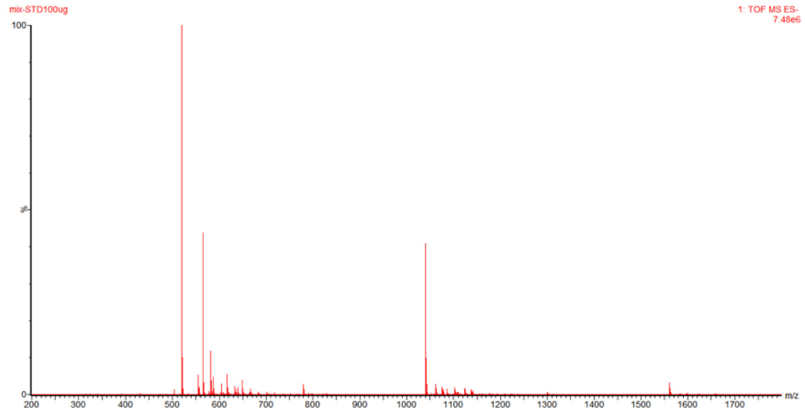

Figure S2 Ion chromatograms of the five main chemical components. A: Deapio Platycodin D; B: Platycodin D; C: Platycodin D_3; D: Platycodin D_2; E: Platycodigenin.

2. Analysis of total RNA integrity

The extracted total RNA was subjected to agarose gel electrophoresis and the results are shown in Figure S3. The 28s band was wider and brighter than the 18s band, confirming that the 28s band was approximately twice as wide and brighter than the 18s band in the RNA composition: the 5s band was lighter in colour, proving that the RNA was not degraded and had good integrity and could be further used for CDNA synthesis.

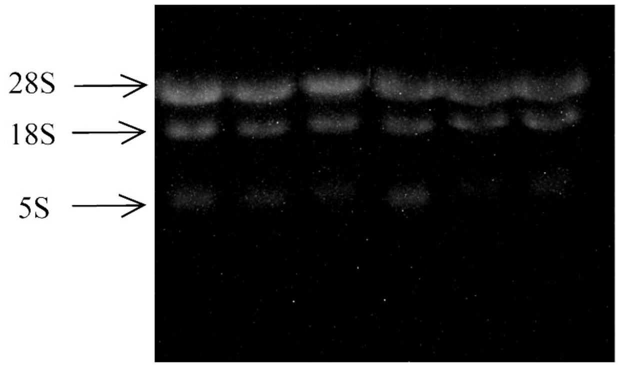

Figure S3 The gel electrophoresis of total RNA
